# Foveal Avascular Zone in Normal Tension Glaucoma Measured by Optical Coherence Tomography Angiography

**DOI:** 10.1155/2017/3079141

**Published:** 2017-12-17

**Authors:** Maja Zivkovic, Volkan Dayanir, Tolga Kocaturk, Marko Zlatanovic, Gordana Zlatanovic, Vesna Jaksic, Marija Radenkovic, Predrag Jovanovic, Sanja Sefic Kasumovic, Mladjan Golubovic, Svetlana Jovanovic

**Affiliations:** ^1^Medical Faculty, Department of Ophthalmology, University of Nis, Nis, Serbia; ^2^Ophthalmology Clinic, Clinical Center Nis, Nis, Serbia; ^3^BATIGOZ Health Group Eye Clinic, Izmir, Turkey; ^4^Department of Ophthalmology, Adnan Menderes University Medical School, Aydin, Turkey; ^5^Medical Faculty, Department of Ophthalmology, University of Belgrade, Belgrade, Serbia; ^6^Eye Clinic “Dr. Sefic”, Sarajevo, Bosnia and Herzegovina; ^7^Center for Anesthesiology and Reanimatology, Clinical Center Nis, Nis, Serbia; ^8^Department of Ophthalmology, Faculty of Medical Sciences, University of Kragujevac, Kragujevac, Serbia

## Abstract

**Aim:**

To measure diameter of foveal avascular zone (FAZ), FAZ area, and vessel density using Optical Coherence Tomography Angiography (OCT-A) in patients with normal tension glaucoma (NTG) and to establish the possible role of OCT-A in diagnosis and follow-up of patients with NTG.

**Methods:**

Twenty-one eyes of 21 patients with NTG and 30 eyes of 30 healthy subjects underwent complete ophthalmic examination as well as OCT-A on ZEISS AngioPlex. 3 × 3 macula scans were used to measure vertical, horizontal, and maximum diameter of FAZ by two graders. Mean values and interobserver variability were analyzed. Image J was used for analysis of FAZ area and vessel density.

**Results:**

Mean vertical diameter (*t* = 5.58, *p* < 0.001), horizontal diameter (*t* = 3.59, *p* < 0.001), maximum diameter (*t* = 5.94, *p* < 0.001), and FAZ area (*t* = 5.76, *p* < 0.001) were statistically significantly enlarged in the NTG group compared to those in the control group. Vessel density (*t* = −5.80, *p* < 0.001) was statistically significantly decreased in the NTG group compared to that in the control group.

**Conclusion:**

OCT-A could have an important role in the future in diagnosis of patients with NTG. In patients with NTG, there is larger FAZ area, while the vessel density is reduced in comparison to the control group.

## 1. Introduction

Normal tension glaucoma (NTG), also called low-tension glaucoma, is a chronic, progressive optic neuropathy with optic nerve deterioration and glaucomatous visual field defects [1]. Values of intraocular pressure (IOP) do not exceed the normal range of 21 mmHg [2]. Vascular dysfunction has been proposed as one of the most important factors in the development and progression of NTG3. This makes the study of blood flow in NTG extremely essential. The mechanisms underlying the abnormal ocular blood flow [3–5] in NTG are still not clear, but the risk factors for glaucomatous optic neuropathy likely include oxidative stress [6], vasospasm [7], and endothelial dysfunction [8].

According to the vascular theory, damage results from low or fluctuating ocular blood flow (OBF) causing ischemia and reperfusion injury in optic nerve head (ONH), respectively [9]. The role of vasospasm and ischemia as the primary cause of such a vascular failure in the systemic micro- and macrocirculation [10] in NTG leads to chronic oxidative stress affecting ONH and ganglion cells.

Several techniques (color Doppler imaging (CDI), scanning laser ophthalmoscopy, fundus fluorescein angiography (FFA), and laser Doppler flowmetry) have been used to measure retrobulbar and intraocular hemodynamics in patients with glaucoma [11]. All the methods currently in use to measure ocular blood flow (OBF) have inherent limitations and measure different aspects of OBF [11].

In the last year, ophthalmology had a revolutionary discovery, Optical Coherence Tomography Angiography (OCT-A). We can measure dimensions of the foveal avascular zone (FAZ) with great reproducibility for the first time [12]. Since vascular theory is one of the proposed theories in pathogenesis of NTG, OCT-A might help us to better understand the pathogenesis of NTG. Until now, few papers have studied peripapillary vessel density in primary open angle glaucoma (POAG), but no paper has studied dimensions of FAZ in patients with NTG [12, 13]. To the best of our knowledge, this is the first study evaluating FAZ in NTG patients using OCT-A.

## 2. Aim of the Paper

The aim of this paper is to measure diameter size of FAZ, FAZ area, and vessel density using OCT-A in patients with NTG and to establish possible role of OCT-A in diagnosis of patients with NTG.

## 3. Methods

This is a cross-sectional case control study. Study was conducted in a group of patients with confirmed NTG and in subjects without glaucoma that were age and gender matched. Twenty-one eyes of 21 patients with NTG and 30 eyes of 30 healthy subjects underwent complete ophthalmic examination as well as OCT-A on Zeiss AngioPlex, CIRRUS HD-OCT Model 5000 instrument (Carl Zeiss Meditec, Dublin, CA). Considering that FAZ can vary in size and shape, control group was established with a bigger number of healthy persons. Ocular examinations were performed in the period from June 2016 to December 2016 at the Ophthalmology Eye Hospital-Clinic Maja, Nis, Serbia. This study was approved by the Ethics Committee from Ophthalmology Eye Hospital-Clinic Maja, Nis, Serbia, following the tenets of the Declaration of Helsinki with informed consent obtained from all the participants. All the patients were with previously diagnosed NTG and complete cardiological, neurological, and rheumatological examination already done, as well as color Doppler imaging. All patients were treated with topical antiglaucoma drug *α*2-agonist brimonidine 0.2% for at least one year and a maximum of two years, without any other topical or systemic medication taken in the last year.

Each patient underwent a complete ophthalmic examination including best corrected visual acuity (BCVA), IOP measurement by Goldman applanation tonometry, gonioscopy, visual field testing using Swedish interactive thresholding algorithm (SITA-standard) central 24-2 Humphrey perimetry (Humphrey field analyzer II, Carl Zeiss Meditec, Dublin, CA, USA), biomicroscopy, fundus examination with a plus 90-diopter lens, multicolor disc photograph, and OCT-A on Zeiss AngioPlex, CIRRUS HD-OCT Model 5000 instrument (Carl Zeiss Meditec, Dublin, CA). Diagnostic criteria for NTG follow the criteria of the European Glaucoma Society [1]: (1) patients older than 35 years, (2) normal IOP without treatment, less than 21 mmHg, (3) optic nerve head damage typical of glaucoma, (4) visual field defects typical of glaucoma, (5) gonioscopy, open anterior chamber angle, and (6) no history of steroid use. Patients with systemic disease with ocular involvement like diabetes and neurological diseases capable of causing visual field loss or optic disc deterioration and other eye disease (except glaucoma), opacification of ocular media, intraocular surgery, refractive errors more than +/− 2 D, and history of ocular trauma were excluded from the study. The data was stratified based on patient Caucasian race.

Inclusion criteria for the control group (CG) were (1) no history or evidence of retinal and eye pathology, (2) no history of intraocular surgery, (3) IOP ≤ 21 mmHg, (4) having to be free of optic disc damage and without any systemic and neurological diseases capable of causing visual field loss or optic disc deterioration, (5) intact neuroretinal rim and normal retinal nerve fiber layer (RNFL) thickness, and (6) normal standard automated perimetry (defined as a glaucoma hemifield test within normal limits and a pattern standard deviation within 95% confidence-interval limits).

OCT angiography was scanned by a 68 kHz Cirrus HD-OCT 5000-based Optical Micro Angiography (OMAG) prototype system. Automated segmentation of the superficial and deep capillary plexus was used for analysis. Images obtained were checked for quality (Signal Strength more than 6/10), as well as absence of artifacts.

**Figure 1:**
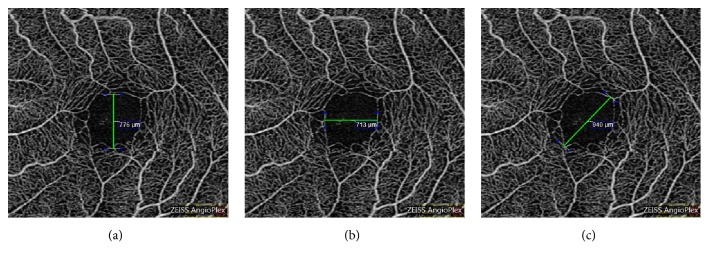
Measurement of (a) vertical, (b) horizontal, and (c) maximum diameter of foveal avascular zone.

Currently, OCT-A Zeiss AngioPlex allows access to 3 × 3, 6 × 6, and 8 × 8 scans. In this study, considering that it was very important to get accurate measurement of FAZ, 3 × 3 scans were exclusively used as they give the best view of the FAZ. We have analyzed the dimensions of FAZ manually by measuring vertical and horizontal diameters and used Image J for analysis of FAZ area and vessel density. Also considering the phenomenon of capillary drop out in patients with NTG, we introduced the concept of maximum diameter that would fit the largest diameter of FAZ for a given FAZ analyzed (Figures 1(a)–1(c)). Two experienced ophthalmologists who were blind to the diagnoses made all the measurements of FAZ diameters (vertical, horizontal, and maximum) in order to determine interobserver variability. Superficial vascular network of angiography was used since it has been proven that there is a high interobserver variability in measuring of the deep vascular network, primarily due to the shadow artifacts caused by vessels of superficial vascular network. Superficial vascular network supplies the superficial structures, namely, RNFL, ganglion cell layer, and inner plexiform layer (IPL), that are affected by glaucoma. Mean values of vertical, horizontal, and maximum diameters were used for comparison.

**Figure 2:**
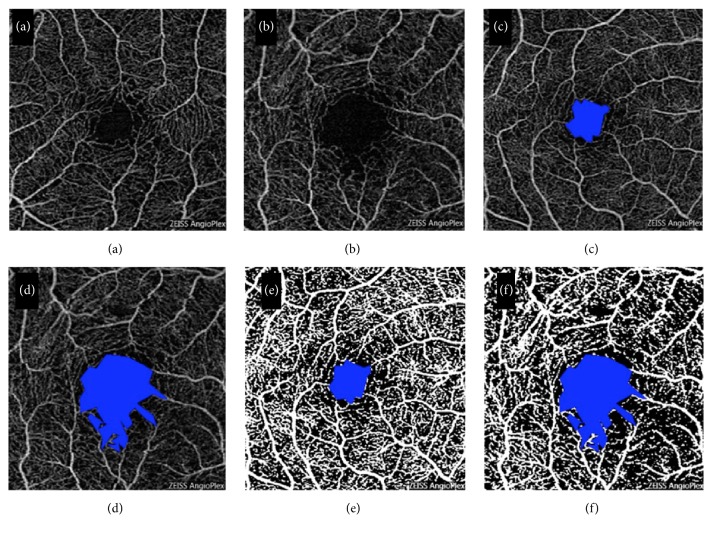
A 3 × 3 macular OCT-A scan of a normal eye (a) and NTG eye (b) is seen. Area of FAZ of the same normal eye (c) and NTG eye (d) as outlined by Image J program and shown in blue. Binarized image for the same normal eye (e) and NTG eye (f) showing vessels in white in order to calculate macular vessel density.

All 3 × 3 OCT-A images were exported from the system as a Joint Photographic Experts Group file into the National Institutes of Health Image J 1.50 (a publically available image processing program developed by National Institutes of Health, Bethesda, Maryland, USA) software. The FAZ area and vessel density were calculated using a previously published method [14]. FAZ area was manually outlined by the polygon selection tool. The dimension of the FAZ was expressed as square millimeters [15]. To calculate vessel density, images were binarized through a threshold strategy similarly to other studies [14, 15]. The image was converted from 8-bit into red green blue (RGB) colour type. The adjusting threshold tool set to mean was applied; the dark-background option was selected. The FAZ area was colored to pure blue. White pixels were considered as vessel, black pixels were considered as background, and blue pixels were automatically excluded from the analysis. Vessel density was expressed as the ratio of vessel pixels to the total area (Figures 2(a)–2(f)).

### 3.1. Statistical Analysis

Statistical analysis was performed with the statistical package SPSS 22 for Windows. Primary data obtained were analyzed by descriptive statistical methods and methods for testing hypotheses. From the descriptive statistical methods, the following were used: measures of central tendency, measures of variability, and structure indicators expressed as a percentage. To determine the normality of distribution, Kolmogorov-Smirnov test was used. To test differences between 2 arithmetic means, the *t*-test for independent samples (Independent-Samples *t*-test) was used. To test differences of arithmetical mean between 2 measurements, *t*-test for dependent samples (Paired-Samples *t*-test) was used. Chi-square test of homogeneity was examined whether the two groups differ according to the proportion of the variable of interest. To test interobserver reliability, double mixed model intraclass correlation coefficient (ICC) was used.

Correlation analysis was performed by measuring the correlation between two variables. The Pearson's correlation coefficient was used to determine the direction and strength of the connection. The conclusion was done at the level of statistical significance of 0.05.

## 4. Results

### 4.1. Patient Demographics and Baseline Characteristics of the Group

NTG group included 21 eyes of 11 female and 10 male patients. Mean age was 70.1 ± 6.4 years (mean ages of women and men were 69.6 ± 6.4 and 70.5 ± 6.7 years, resp.). Control group consisted of 30 eyes of 16 female and 14 male subjects. Mean age was 69.5 ± 5.4 years (mean ages of women and men were 71.6 ± 5.2 and 67.0 ± 4.6 years, resp.). There was no statistically significant difference between the NTG and control groups with respect to gender proportion (*χ*^2^ = 0.46, *p* = 0.61) and age (*t* = 0.40, *p* = 0.70). In the NTG and control groups, there was no statistically significant difference in the participation of the left and right eyes (*χ*^2^ = 0.00, *p* = 1.00) (Table 1).

Statistically significant difference was not observed between the two measurements done within NTG and control groups, respectively, for vertical (*t* = −1.32, *p* = 0.238; *t* = −1.90, *p* = 0.074), horizontal (*t* = −1.19, *p* = 0.124; *t* = −1.92, *p* = 0.069), and the maximum diameters (*t* = −0.78, *p* = 0.422; ⁡*t* = −1.79, *p* = 0.086) (Table 2).

Measurements done by two independent observers in the NTG and control groups, respectively, for vertical (*r* = 0.99, *p* < 0.001; *r* = 0.99, *p* < 0.001), horizontal (*r* = 0.77, *p* < 0.001; *r* = 0.99, *p* < 0.001), and the maximum diameter (*r* = 0.99, *p* < 0.001; *r* = 0.99, *p* < 0.001) were significantly positively correlated.

Mean foveal avascular zone vertical diameter (*t* = 5.58, *p* < 0.001), horizontal diameter (⁡*t* = 3.59, *p* < 0.001), maximum diameter (*t* = 5.94, *p* < 0.001), and foveal avascular zone area (*t* = 5.76, *p* < 0.001) in 3 × 3 macula superficial scan were all statistically significantly enlarged in NTG group compared to those in the control group. Vessel density (*t* = −5.80, *p* < 0.001) was statistically significantly decreased in the NTG group compared to that in the control group (Table 3).

Interobserver agreement was high for all measurements (vertical diameter, horizontal diameter, and maximum diameter) (Table 4).

## 5. Discussion

Vascular factors play a significant role in the development of NTG. Blood supply of the eye is mainly from the ophthalmic artery (OA), a branch of the internal carotid artery (ICA). The OA gives rise to ciliary arteries, which supply the choroid, outer retina, and ONH, and the central retinal artery (CRA) that supplies inner retina. Branches of CRA branch out on the RNFL level making up the superficial plexus within the ganglion cell layer while the deeper branches reach out to the inner nuclear layer and provide deep plexus [8, 10, 11].

For retrobulbar circulation monitoring, currently the most widely used method is CDI. CDI is a perfect tool to assess the large ophthalmic vessels, such as the OA, CRA, and the short posterior ciliary arteries (SPCAs) [16]. The mean velocity and the resistivity index (RI) are the two important parameters that can be followed, except that RI does not accurately correlate with the resistance offered by the ophthalmic vessels [17, 18]. Kocaturk et al. reported increased vascular resistance in OA in pseudoexfoliation glaucoma [19]. Many studies demonstrated that the blood flow resistance was increased and blood flow velocities were decreased in the OA and SPCAs of patients with POAG, pseudoexfoliation syndrome, and NTG [9, 17–19]. Reduced blood flow velocities and increased RI in retrobulbar vessels in patients with NTG may explain the reason for the ONH fluorescein filling defects and capillary loss of the ONH of these patients [18].

Since it is proven that there are changes at the level of OA, CRA, and SPCAs, the main arteries that supply the eye with blood, great importance is given in finding adequate methods that will examine the most credible changes in intraocular blood vessels. Numerous studies have followed the retinal arteriovenous passage (AVP) times and the perfusion of retinal and choroidal microvascular beds with fluorescein angiography and found that the AVP was prolonged in NTG patients [20]. Arend et al. examined whether a macular capillary density reduction and/or peripapillary diameter changes account for the pathological retinal circulation in patients with NTG [21]. They speculated that pathological alterations of arterial or venous diameter or macular vasculature might occur in NTG, causing gliosis-like repair mechanisms, or that retinal capillary density is reduced as a sign of ischaemic disease. Capillary dropout or vasoconstriction of the retinal vasculature could contribute to increased resistance. In the study, they used digital scanning laser fluorescein angiography (Rodenstock Instruments, Ottobrunn, Germany) and the angiograms were recorded on NTSC Sony videotapes. Their results showed that the AVP time of NTG patients was significantly prolonged when compared with healthy subject data, but morphological data of FAZ and perifoveal intercapillary areas did not differ from healthy subjects; neither did peripapillary arterial and venous diameter measurements [21].

In recent years, the measurement of ocular blood flow has become easier and noninvasive owing to the development of OCT-A. OCT-A enables us to measure the dimension of FAZ, capillary-free area in central macula with high accuracy. Up to now, there was not any information in the literature on FAZ area and macular vessel density at NTG measuring with OCT-A, although there are many papers related to the peripapillary and macular vessel density in healthy persons [22, 23] and in patients with the POAG. Yarmohammadi et al. have found that reduced peripapillary and macular vessel density were detectable in the perimetrically intact hemiretinae of glaucoma eyes with a single-hemifield defect [24]. Their conclusion was that OCT-A potentially shows promise for identifying focal glaucomatous damage before VF defects are detectable.

Numerous studies using OCT-A in patients with open angle glaucoma have demonstrated reduced ONH and peripapillary vessel densities in patients with glaucoma [25–27]. Also many studies have demonstrated that peripapillary angioflow density shows strong correlation with the RNFL thickness [26–28].

There are only few papers that were done with NTG patients, but the authors followed only peripapillary vessel density, not macular vessel density. Shin et al. demonstrated that peripapillary vessel density maps of superficial and deep retinal layers were significantly reduced at the 7 and 11 o'clock positions in glaucomatous eyes [29]. Scripsema et al. compared perfused peripapillary capillary densities in POAG, NTG, and normal patients using OCT-A and showed that POAG and NTG patients had a reduction of perfused capillaries that progressed in size when comparing early, moderate, and severe glaucoma groups [30]. Bojikian et al. investigated optic disc perfusion differences in healthy subjects, POAG, and NTG and showed that optic disc perfusion was significantly reduced in POAG and NTG groups compared to normal controls, but no difference was seen between POAG and NTG groups with similar levels of VF damage [20]. Igarashi et al. published that flow density and the disappearance angle of the radial peripapillary capillaries were significantly and independently correlated with glaucoma-related functional and morphological changes in the optic nerve in POAG and NTG [31].

Our study is the first one that follows macular vessel density and FAZ area in NTG. In our study, larger FAZ areas with reduced vessel density were found in NTG patients. It would be possible to define an exact value of FAZ area to support the diagnosis of NTG or other types of glaucoma with larger number of patients. These vascular parameters are gradually becoming important in terms of diagnosis, progression, and treatment options in the management of the patients with glaucoma.

As far as we know, this is the first study that will shed light on further investigation about FAZ in patients with NTG. Further investigations related to the FAZ and peripapillary and macular vessel density are needed with larger numbers of patients in order to determine the role of OCT-A in glaucoma.

## 6. Conclusion

OCT-A could have an important role in the future in diagnosis of patients with NTG as well as in understanding the pathophysiology of NTG. We have shown that, for the first time in the literature, NTG patients have larger FAZ area and reduced macular vessel density compared to the control group.

## Figures and Tables

**Table 1:** Demographic and baseline characteristics.

	NTG group (*n* = 21)	Control group (*n* = 30)	*P* value
Number of eyes (patients)	21* *	30	
Eye (left/right)	11/10	15/15	1.00^a^
Men age (SD), years	70.1 (6.4)	69.5 (5.4)	0.70^b^
Gender (female/male)	11/10* *	16/14	0.61^a^

^a^ *χ* ^2^-test, ^b^*t*-test, NTG = normal tension glaucoma, and SD = standard deviation.

**Table 2:** Characteristics of vertical, horizontal, and maximum diameters measured by two independent observers.

	1st observer *x* ± SD (min-max)	2nd observer *x* ± SD (min-max)	*P* value	*r*
NTG group				
Vertical diameter	648.98 ± 123.76 (510–1000)	650.33 ± 118.87 (525–989)	0.238^a^	0.99^b*∗*^
Horizontal diameter	631.50 ± 64.44 (520–840)	630.92 ± 69.89 (541–829)	0.124^a^	0.77^b*∗*^
Maximum diameter	731.45 ± 111.02 (624–1055)	732.23 ± 110.13 (608–1034)	0.422^a^	0.99^b*∗*^
Control group				
Vertical diameter	512.02 ± 89.45 (336–706)	513.43 ± 84.23 (353–690)	0.074^a^	0.99^b*∗*^
Horizontal diameter	546.54 ± 86.65 (427–804)	548.68 ± 83.35 (442–799)	0.069^a^	0.99^b*∗*^
Maximum diameter	592.11 ± 95.32 (440–802)	582.45 ± 90.24 (470–819)	0.086^a^	0.99^b*∗*^

^a^ *t*-test, ^b^*r* = Pearson's correlation coefficient, SD = standard deviation, and NTG = normal tension glaucoma; ^*∗*^*p* < 0.001.

**Table 3:** Characteristics of the average value of vertical, horizontal, and maximum diameter, foveal avascular zone area, and vessel density in NTG and control group.

Average between 2 measurements	NTG group *x* ± SD (min-max)	Control group *x* ± SD (min-max)	*P* value
Vertical diameter	649.43 ± 112.55 (517.5–994.5)	512.23 ± 85.90 (344.5–697.0)	<0.001^a^
Horizontal diameter	630.34 ± 63.10 (530.5–834.5)	559.77 ± 83.52 (434.5–801.5)	<0.001^a^
Maximum diameter	731.45 ± 112.96 (616.0–1044.5)	586.83 ± 88.39 (455.5–810.5)	<0.001^a^
Foveal avascular zone area	0.412 ± 0.060 (0.295–0.472)	0.292 ± 0.048 (0.178–0.399)	<0.001^a^

Vessel density	39.55 ± 3.81 (35.40–41.90)	46.21 ± 3.36 (43.10–50.80)	<0.001^a^

^a^ *t*-test, NTG = normal tension glaucoma, *x*  = mean, and SD = standard deviation.

**Table 4:** Interobserver reliability.

	NTG group (*n* = 21) ICC (95% CI)	Control group (*n* = 30) ICC (95% CI)
Vertical diameter	0.996 (0.991–0.999)	0.994 (0.990–0.997)
Horizontal diameter	0.989 (0.974–0.995)	0.994 (0.988–0.996)
Maximum diameter	0.996 (0.991–0.998)	0.996 (0.992–0.998)

NTG = normal tension glaucoma, ICC = intraclass correlation coefficient, and 95% CI = 95% confidence interval.
